# Ultrasensitive tumour‐agnostic non‐invasive detection of colorectal cancer recurrence using ctDNA methylation

**DOI:** 10.1002/ctm2.1015

**Published:** 2022-09-14

**Authors:** Yu Xiao, Xiaodong Wang, Hong Weng, Zhao Ding, Kaiyu Qian, Wan Jin, Sen Lu, Lingao Ju, Zhiwen He, Gang Wang, Xiaoyu Xie, Dongmei Liu, Zhou Fan, Kai Wu, Sheng Li, Huanhuan Guo, Guofeng Qian, Wei Jiang, Yunji Leng, Junpeng Zhao, Xinyue Cao, Minsheng Peng, Congqing Jiang, Li Li, Yi Zhang, Xinghuan Wang

**Affiliations:** ^1^ Department of Biological Repositories Zhongnan Hospital of Wuhan University Wuhan China; ^2^ Human Genetic Resource Preservation Center of Hubei Province Wuhan China; ^3^ Department of Gastroenterology Surgery West China Hospital of Sichuan University Chengdu China; ^4^ Center for Evidence‐Based and Translational Medicine Zhongnan Hospital of Wuhan University Wuhan China; ^5^ Department of Urology Zhongnan Hospital of Wuhan University Wuhan China; ^6^ Department of Colorectal and Anal Surgery Zhongnan Hospital of Wuhan University Wuhan China; ^7^ Clinical Center of Intestinal and Colorectal Diseases of Hubei Province Wuhan China; ^8^ Euler Technology ZGC Life Sciences Park Beijing China; ^9^ Department of Colorectal and Anal Surgery The First Affiliated Hospital of Zhejiang University Hangzhou China; ^10^ Wuhan Research Center for Infectious Diseases and Cancer Chinese Academy of Medical Sciences Wuhan China; ^11^ Department of Endocrinology The First Affiliated Hospital of Zhejiang University Hangzhou China; ^12^ Medical Research Institute Wuhan University Wuhan China; ^13^ Clinical Trial Center Zhongnan Hospital of Wuhan University Wuhan China; ^14^ State Key Laboratory of Genetic Resources and Evolution Kunming Institute of Zoology, Chinese Academy of Sciences Kunming China; ^15^ University of Academy of Sciences Kunming College of Life Science Kunming China

1

Dear Editor,

Colorectal cancer (CRC) is the third most common cancer (10.0%) and the second leading cause of cancer‐related deaths (9.4%) worldwide.^1^ Here we show cancer‐derived DNA methylation (DNAm) enables sensitive and specific non‐invasive diagnosis and monitoring of CRC.

Cell‐free DNA (cfDNA) containing tumour‐derived biomarkers provide a promising avenue for non‐invasive detection of minimal residual disease (MRD) after curative‐intent surgery.^2^ Tumour‐derived DNA, or circulating tumour DNA (ctDNA), could be detected by sequencing cfDNA from post‐surgery or adjuvant therapy blood.^3^ We performed a prospective, observational, and multi‐centre cohort study (Cancer HALLmark Epigenetics aNd GEnetics in CRC, *Challenge‐CRC*) with 280 CRC patients (Stages I–IV) to assess the ability of a tumour‐agnostic ctDNA assay to identify patients with MRD that would ultimately recur. Pre‐ and post‐operative cfDNA were obtained from the patients. Somatic mutation and DNAm were sequenced and analysed. Statistics is performed to compare the performance to detect MRD from somatic mutation and DNAm (Figure 1).

**FIGURE 1:**
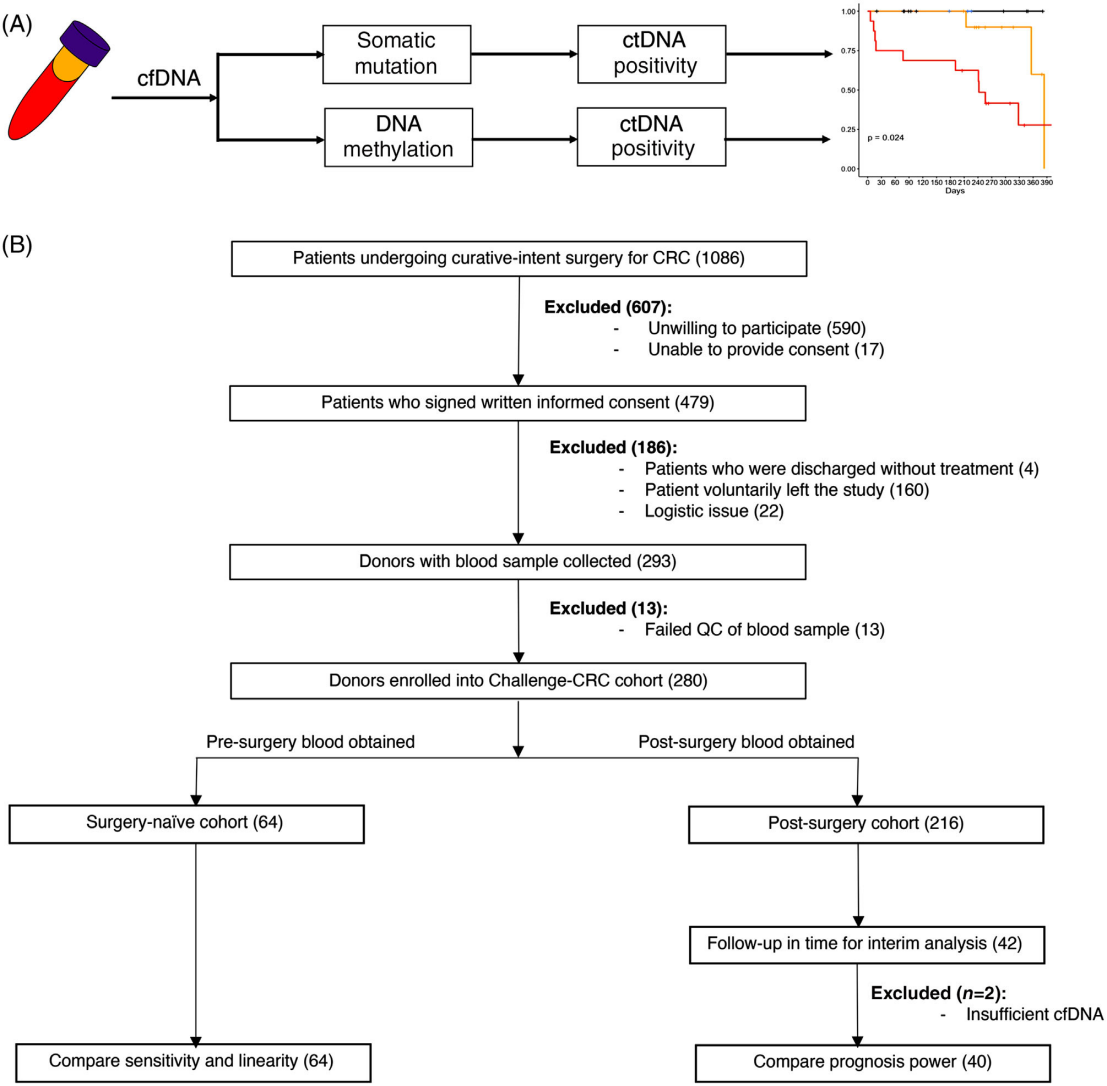
Overview of the study. (A) Schematic diagram of the study. Cell‐free DNA (cfDNA) was extracted from peripheral blood and undergone parallel sequencing of somatic mutation or DNA methylation. Cancer‐derived somatic mutation, or cancer‐associated DNA methylation, were extracted from the sequencing data. Tumour fraction and malignancy state were derived from the extracted signal. Tumour fractions derived from somatic mutation or DNA methylation were compared. Post‐surgery DNA methylation was used to predict recurrence‐free survival. (B) Enrolment flowchart of the study.

In this interim analysis, patients were divided into a surgery‐naïve group (*n* = 64) and a post‐curative‐intent‐surgery group (*n* = 40) (Tables S1 and S2). For the surgery‐naïve group, 64 surgery‐naïve, initially diagnosed, untreated CRC patients were enrolled. Enrolled patients include 37 (57.8%) colon cancer and 27 (42.2%) rectal cancer of clinical Stages I (3.1%, 2/64), II (20.3%, 13/64), III (34.4%, 22/64) and IV (42.2%, 27/64). Overall, 82.8% of patients (53/64) are lymph‐node metastasis‐free, and 68.8% (44/64) patients are distal metastasis‐free. For the post‐surgery group, enrolled patients include 10 (25.0%) colon cancer and 30 (75.0%) rectal cancer of clinical Stages II (10.0%, 4/40), III (27.5%, 11/40) and IV (62.5%, 25/40). Overall, 37.5% of patients (15/40) are lymph‐node metastasis‐free, whereas 62.5% (25/40) patients are with N1/N2. Overall, 22.5% (9/40) patients received neoadjuvant therapy and 67.5% (27/40) received adjuvant therapy. Overall, 22.5% (9/40) patients had surgery alone with no neoadjuvant or adjuvant therapy. Patients were followed up with a median of 239 days post‐operatively. Overall, 32.5% (13/40) patients recurred with a median time to recurrence from surgery of 214 days (range, 6–384). Blood was drawn a median of 14 days (range, 6–307) post‐operatively.

In pre‐operative blood cfDNA, with a limit‐of‐detection of 0.5% variant allelic frequency (VAF), 50.0% (32/64) patients were somatic mutation positive (Tables S3–S5). As expected, detected putative tumour‐specific mutations include pathogenic mutations in TP53, KRAS, APC and SMAD4 (Figure 2A). Structural variants derived gene fusions of FGFR1 or NTRK1 were detected in two cases. These results closely resemble the reported mutation landscape in CRC.^4^

**FIGURE 2:**
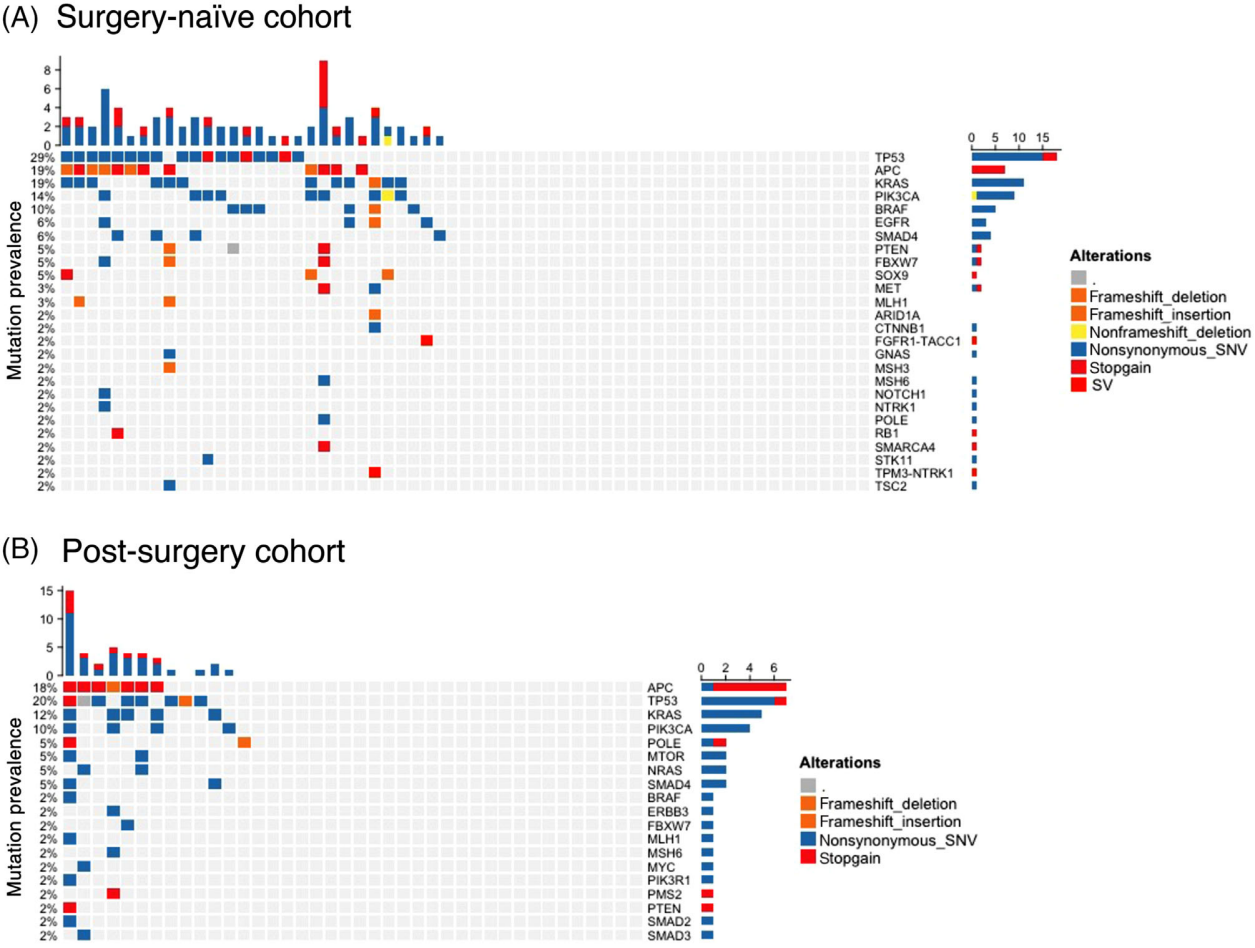
Somatic hotspot mutations. (A) Somatic hospot mutation cascade plot in surgery‐naïve, pre‐surgery blood. (B) Somatic hotspot mutation cascade plot in post‐surgery blood.

Mean frequency of CRC‐specific DNAm haplotypes, or the mean VAF of tumour‐specific somatic mutation (including structural variation) (Figure 2B), were independently used to derive tumour fraction (TF) in cfDNA. In samples with positive somatic mutation detected, methylation‐derived TF linearly correlates with mutation‐derived TF (adj. *R* ^2^ = 0.9074, *p* < 2.2e − 16, Figure 3A). Furthermore, many samples without any detected somatic mutation were positive for methylation‐derived TF (Figure 3A,B). Combining tumour‐derived DNAm signal with tumour‐associated, immune‐related DNAm signal, age‐related DNAm signal and cfDNA fragment size information further improved the performance of cfDNA methylation. Here, by leveraging DNAm correlating with multiple, orthogonal biological features, we constructed the MAFIT score to report an overall tumour‐associated DNAm level in the sample (see the Supporting Information section). Overall, when considering both tumour‐derived and tumour‐associated, immune‐related signals, DNAm was positive in 78.1% (50/64) of samples, whereas cfDNA mutation was positive in 50.0% (32/64) of samples. All samples positive with somatic mutation were also positive for DNAm, and DNAm additionally detected ctDNA signal in 56.3% (18/32) somatic‐mutation‐free samples. We conclude that compared to cfDNA mutation, DNAm is more sensitive for detecting presence of tumour (detection odds ratio 3.53 (1.56–8.36), *p* = 0.001595, Fisher's exact test).

**FIGURE 3:**
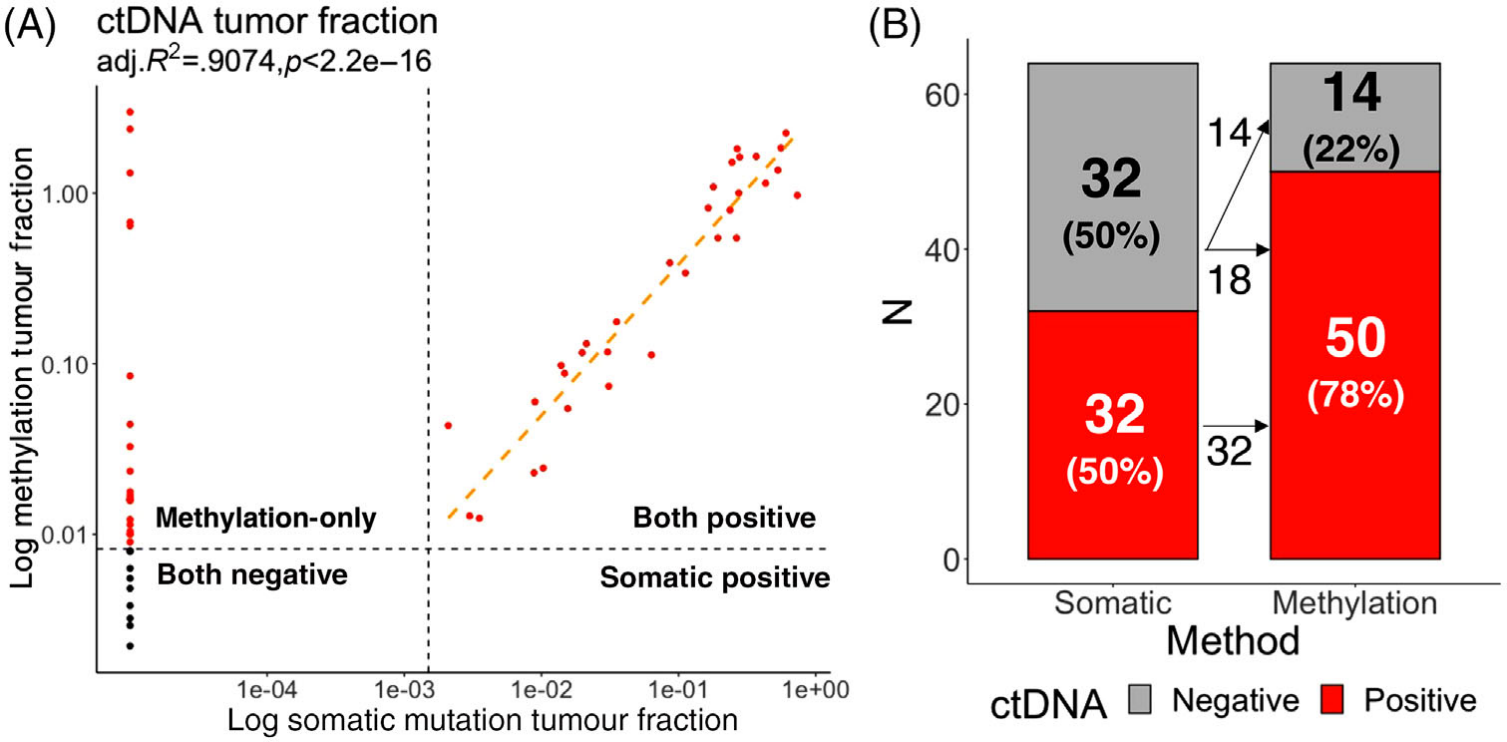
Comparison between somatic mutation and DNA methylation‐derived tumour fraction in the surgery‐naïve cohort. (A) Scatter plot of tumour fraction in circulating tumour DNA (ctDNA)‐positive samples. Somatic mutation‐derived tumour fraction is on *X* axis, and DNA methylation‐derived tumour fraction is on the *Y* axis. (B) Number and ratio of ctDNA positivity with somatic mutation or DNA methylation alone, together with the flow of donors between tests, in pre‐surgery blood from the surgery‐naïve cohort.

In post‐operative blood cfDNA from 40 patients received curative‐intent surgery, somatic mutation was positive in 47.5% (19/40) patients (Tables S4–S6), whereas DNAm was positive in 67.5% (27/40) patients (Figure 4A,C). In analysing progression‐free survival (Figure 4B,D), 100% (13/13) of patients who are negative for DNAm, regardless of somatic mutation, were recurrence‐free during the whole observation period (12 months). Tumour recurrence was found in 27.3% (3/11) of patients who are methylation‐positive but somatic‐mutation‐negative with a median RFS of 384 days (95% CI: 356‐Inf), or 62.5% (10/16) of patients who are double‐positive for somatic mutation as well as DNAm, with a median RFS of 242 days (95% CI: 77‐Inf.). Compared with somatic mutation predicted ctDNA positivity, methylation‐derived ctDNA positivity has similar PPV (48.1% vs. 52.6%, *p* = 1, Fisher's exact test) but higher NPV (100% vs. 85.7%, *p* = 0.2701, Fisher's exact test). Overall, the cfDNA methylation assay shows a sensitivity of 100% and a specificity of 48.1%, whereas the cfDNA somatic mutation assay shows a sensitivity of 76.9% and a specificity of 66.7%. We conclude that methylation‐derived ctDNA positivity was highly predictive for tumour recurrence and outperforms mutation‐derived ctDNA positivity (*p* = 0.024, log‐rank test) in our period of observation.

**FIGURE 4:**
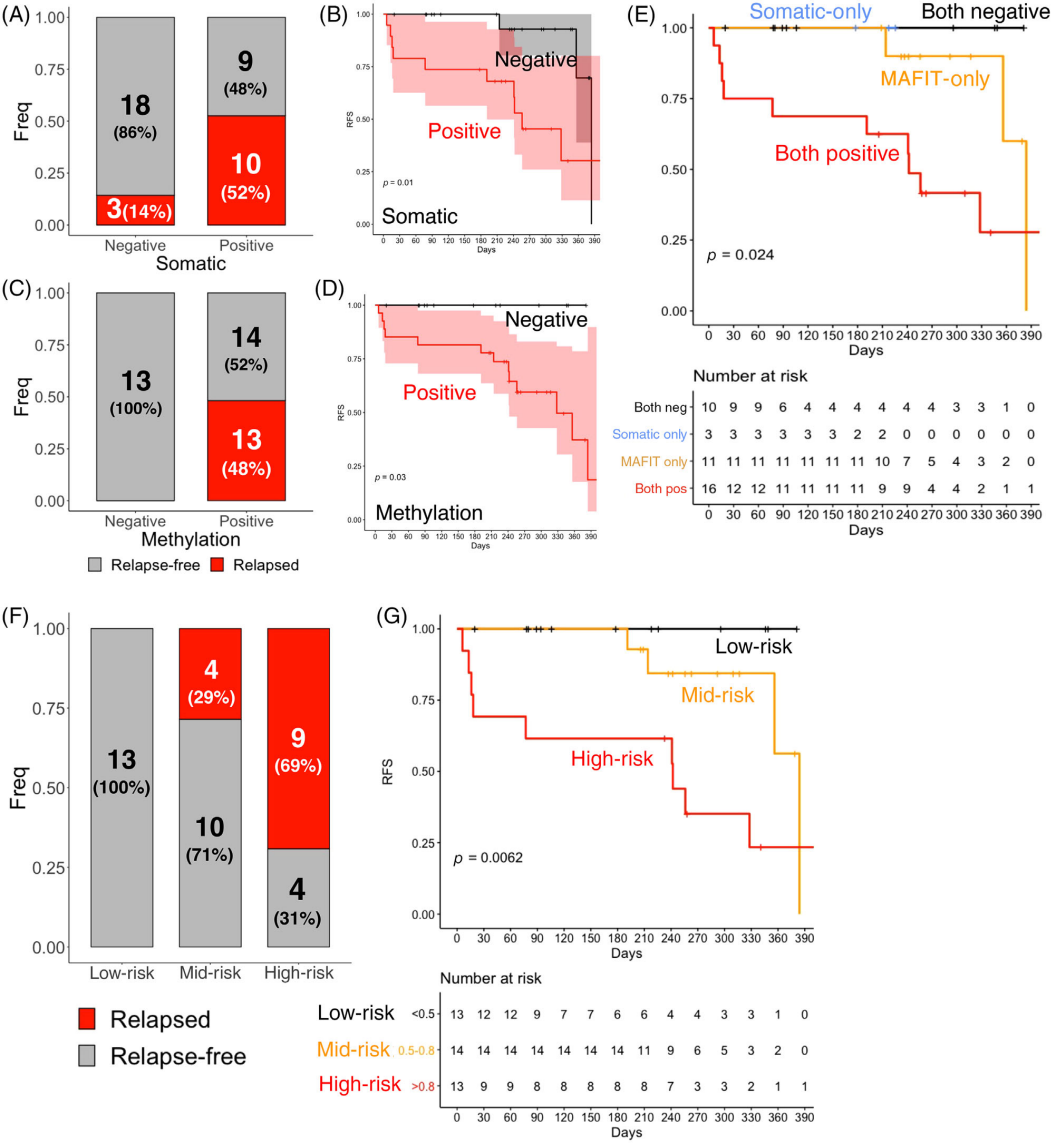
Post‐surgery cell‐free DNA (cfDNA) methylation outperforms cfDNA somatic mutation to predict recurrence. (A) Ratio of circulating tumour DNA (ctDNA) positivity with somatic mutation in post‐surgery blood from the post‐surgery cohort, segregated by disease recurrence. (B) Survival curve of patients stratified by ctDNA positivity predicted by somatic mutation. (C) Ratio of ctDNA positivity with DNA methylation in post‐surgery blood from the post‐surgery cohort, segregated by disease recurrence. (D) Survival curve of patients stratified by ctDNA positivity predicted by methylation. (E) Survival curve of patients stratified by ctDNA positivity predicted by combining somatic mutation and methylation. (F) Recurrence rate in patients of different risks (high‐risk: MAFIT > 0.8; mid‐risk: MAFIT between 0.5 to 0.8 ; low‐risk: MAFIT <0.5). (G) Survival curve of patients of different risk groups, in the post‐surgery cohort.

Patients were grouped into risk strata according to their DNAm level (MAFIT score) in post‐operative blood cfDNA as low‐ (<0.5), mid‐ (between 0.5 and 0.8) and high‐ (>0.8) risk. Overall, 100% low‐risk group patients were progression‐free within 400 days, whereas the medium survival days was 384 days for mid‐risk group (28.6% recurred, 4/14) and 256 days for high‐risk group (69.2% recurred, 9/13) (Figure 4F,G). We conclude that cfDNA methylation is capable of stratifying patients into risk groups with significant different risk of recurrence (Figure 4G and *p* = 0.0062, log‐rank test).

DNAm events predicting CRC have been reported in many studies and applied in several clinical practices for diagnosis and prognosis, for example, detecting methylated SEPT9 DNA in blood, or SDC2/BMP3/NDRG4 DNAm in faecal samples.^5^, ^6^ Diagnosis assays employing a genomic‐epigenomic combined approach to detecting both tumour‐specific somatic mutation (such as KRAS) and immune‐cell‐specific methylation (such as NDRG4)^7^ could enhance overall specificity of the assay and helped to discriminate between benign neoplasm and true malignancy, and monitor MRD.^8^

A major problem of all state‐of‐the‐art MRD detection assay is sensitivity, that is, recurrence in the ctDNA‐negative population. Here we found that cfDNA methylation‐derived TF is linearly correlated with somatic mutation‐derived TF. Furthermore, ctDNA positivity derived by methylation outperformed somatic mutation in terms of predicting recurrence in our period of observation. Superior performance of ctDNA by methylation could be due to two reasons: First, ctDNA detection by methylation is only cell‐type specific and independent from the individualized mutation profile of each tumour, helping to normalize tumour‐specific signal and to ‘rescue’ the detective power for tumours without canonical hotspot or targetable mutations. For example, certain tumour with a novel fusion driver or a DNAm driver (GIST with SDHC germline mutation) could be somatic mutation‐free.^9^ Second, cfDNA methylation carried non‐tumour‐derived, tumour‐associated signals such as remote immune cell activation to enhance detection sensitivity.^10^

The power of our conclusion is limited by a small number of patients with relatively short follow‐up in this interim analysis. Additionally, such short follow‐up has made the post‐surgery cohort does not fully resemble pre‐surgery cohort, which might impact the translation of prediction accuracy of DNAm for tumour load in cfDNA from the pre‐surgery data towards post‐surgery data. Long‐term follow‐up results from the full cohort would be reported in the future, and we expect that these limitations could be fully answered. As the study is carried out during the COVID‐19 pandemic, blood collection times of the patients were not strictly restricted. However we did not notice any correlation between blood sampling time and tumour positivity detection by either somatic mutation or DNAm in our data. Furthermore, as we directly compared paired somatic mutation and DNAm sequencing results from the same blood draw, we did not consider the sampling time affects the power of this study.

In conclusion, we found that DNAm on colorectal‐cancer‐specific DMR based on blood cfDNA sequencing enables an accurate detection of potential relapse of CRC. In the future, it is possible that cfDNAm test of pre‐surgery blood could help one to distinguish between benign disease and truly malignant CRC, to reduce the unnecessary colonoscopy and even unnecessary surgery. Furthermore, post‐surgery blood DNAm signature detects residual disease with high sensitivity to help in suggesting surveillance protocol and indicating time interval of follow‐up or even guiding therapeutic interventions.

## CONFLICT OF INTEREST

The authors declare that they have no conflict of interests.

## CONSENT FOR PUBLICATION

All the authors consent for publication.

## Supporting information

Supporting Information

Supporting Information

Supporting Information

Supporting Information

Supporting Information

Supporting Information

Supporting Information
