# Supplementary material for: Ultrasensitive tumour‐agnostic non‐invasive detection of colorectal cancer recurrence using ctDNA methylation
Source: Clin Transl Med. 2022 Sep 14;12(9):e1015. doi: 10.1002/ctm2.1015 (PMC9473485; doi:10.1002/ctm2.1015)
Supplement: Supplementary file 1 — Supporting Information [file CTM2-12-e1015-s005.docx]

**Table S1. Clinical characteristics of the patients.**

|  | **Surgery-naïve** | **Post-surgery** | **Overall** |
| --- | --- | --- | --- |
|  | **(n=64)** | **(n=40)** | **(n=104)** |
| **Sex** |  |  |  |
| F | 28 (43.8%) | 15 (37.5%) | 43 (41.3%) |
| M | 36 (56.3%) | 25 (62.5%) | 61 (58.7%) |
| **Age** |  |  |  |
| Median [Min, Max] | 58.0 [19.0, 83.0] | 54.0 [32.0, 77.0] | 55.0 [19.0, 83.0] |
| **Position** |  |  |  |
| Colon | 37 (57.8%) | 10 (25.0%) | 47 (45.2%) |
| Rectal | 27 (42.2%) | 30 (75.0%) | 57 (54.8%) |
| **T stage** |  |  |  |
| 1 | 2 (3.1%) | 0 (0%) | 2 (1.9%) |
| 2 | 13 (20.3%) | 4 (10.0%) | 17 (16.3%) |
| 3 | 22 (34.4%) | 11 (27.5%) | 33 (31.7%) |
| 4 | 27 (42.2%) | 25 (62.5%) | 52 (50.0%) |
| **N stage** |  |  |  |
| 0 | 53 (82.8%) | 15 (37.5%) | 68 (65.4%) |
| 1 | 7 (10.9%) | 12 (30.0%) | 19 (18.3%) |
| 2 | 4 (6.3%) | 13 (32.5%) | 17 (16.3%) |
| **M stage** |  |  |  |
| 0 | 44 (68.8%) | 20 (50.0%) | 64 (61.5%) |
| 1 | 20 (31.3%) | 20 (50.0%) | 40 (38.5%) |
|  |  |  |  |
