# Supplementary material for: Ultrasensitive tumour‐agnostic non‐invasive detection of colorectal cancer recurrence using ctDNA methylation
Source: Clin Transl Med. 2022 Sep 14;12(9):e1015. doi: 10.1002/ctm2.1015 (PMC9473485; doi:10.1002/ctm2.1015)
Supplement: Supplementary file 7 — Supporting Information [file CTM2-12-e1015-s004.doc]

**Supplementary Text**

**Supplementary Discussion**

**Minimal residual disease (MRD) detection in solid tumor and CRC**

Minimal residual disease (MRD) is a concept that first introduced to oncology from leukemia field. The definition of MRD describes a quantitative measurement of residual disease after treatment -- in pre-molecular medicine era, a ‘complete remission’ of leukemia could be achieved by aggressive chemotherapy, with subsequent relapse. MRD defines a way to quantitatively describe the dormant, persistent tumor clone that escaped curative-intent treatment and eventually would lead to future relapse in such context. The migrative nature of hematological neoplasm makes them detectible by fluorescence activated sorting cytometry (FACS), fluorescent in situ hybridization (FISH), or genotyping from circulating blood cells or bone marrow samples.

MRD for solid tumors is similar defined. However, detecting MRD for solid tumors is less straightforward compared to hematological malignancies. The main reason for this is that MRD of solid tumor could exist in forms that not readily assessable cellularly. For example, solid tumor MRD could be tiny tumor clone-lets that seeds to distal organ or lymph node. The extremely limited size of these residual tumor is prohibitive for imaging-based or immunology-based detection methods.

By the transformative technology revolution, a number of techniques has been developed to detect solid tumor MRD. This is brought to light by combination of two powerful molecular biology innovations: the ‘liquid biopsy’, and next generation high-throughput sequencing. Circulating DNA is firstly shown in the blood of systemic lupus erythematosus patients[^1^](#_ENREF_1), and more so in cancer patients[^2-4^](#_ENREF_2). The logic behind such phenomenon is that dying tumor cells release their DNA and RNA into blood, which could be detected by molecular biology assays.

The first developments of liquid biopsy, applied to tumor patients, were mostly tumor-informed approaches, with ddPCR or qPCR to detect known tumor somatic mutations from cell-free DNA (cfDNA). However, these approaches mostly suffer from their sensitivity, by two reasons: firstly, private somatic mutations dominate the mutation profiles of each tumor, and any single somatic DNA mutation cannot detect 100% of patients; secondly, the signal-noise ratio of ddPCR or qPCR is usually at the higher end of 0.1%. Since the cfDNA amount in 10ml of peripheral venous drain blood is usually in the range of 10-15ng, this translates into a 3,000-4,500 copies of genome equivalent. Said so, the fraction of tumor-derived DNA in cfDNA from a tumorlet with 1cm diameter is about 0.007%[^7^](#_ENREF_7), which is not only below the SNR of abovementioned assays, but well beyond the lowest range of detectible signal from 10ml blood: for any single DNA mutation, the chance of detecting a 0.007% mutation in 3,000 copies of genome is rare, if not zero.

The introduction of next-generation sequencing in the field greatly enhanced the detection power of liquid biopsies, by enabling two important features: first, an enhanced signal-noise ratio from ultradeep, error-corrected sequencing[^8-13^](#_ENREF_8); second, a unique capability to engage multiple analytic targets from the very limited starting pool of cfDNA, by firstly analyzing tumor-specific private mutations then design bespoke targeted sequencing panels/amplicon sets to simultaneously interrogate these loci. Applying NGS-based MRD to colorectal cancer (CRC), in particular, predicts clinical relapse with ~8months lead time compared to imaging- and blood analytic based follow-up. A randomized, prospective clinical trial has shown that post-operative MRD negativity predicts void clinical benefit of adjuvant chemotherapy, enabling to avoid unnecessary chemotherapy for these MRD-negative patients[^25^](#_ENREF_25). These prior works firmly established the clinical utility of MRD in clinical management of CRC patients.

**Theoretical considerations on tumor-associated DNA methylation (DNAm) signals**

The ‘bespoke’ or tumor-informed approach of MRD detection has its unique strength: it is cost-effective (once established for the patient), and very sensitive. However, it requires complex workflow: first, a genome-wide mutation profile must be established by sequencing the tumor as well as an appropriate germline control (such as blood mononuclear cell genomic DNA). Subsequently, a targeted assay such as amplicon or targeted-capture panel must be designed for these patient-private mutations, experimentally validated, then applied to the subsequent MRD samples. The workflow is not only tedious, but also subjected to many realistic limitations. For example, the assay would require at least 20 tumor-specific somatic mutations for reliable detection of MRD – which is not always feasible, based on the difficulty of panel design as well as the nature of tumor. Furthermore, in a clinical setting, it is not always possible to obtain the tumor-specific mutation profile simply because the resected tumor would be unavailable.

Several methods have been established to detect pan-tumor molecular features instead of private somatic mutations. These include tumor-specific cfDNA fragment size[^26-28^](#_ENREF_26), tumor-specific cfDNA fragment end signature, and tumor-specific DNA methylation[^31^](#_ENREF_31). In these assays, cancer-associated molecular features are scattered across the genome and exhibit uniform change (at least within the same class of biomarker), enabling at least ~1,000x sensitivity compared to point mutations, in theory. However, the signal-noise ratio of these ‘pan-cancer epigenomic features’ are considerably lower compared to somatic mutation sequencing, resulting in low specificity especially for applications such as MRD or early screen of tumor, where NPV is a very useful parameter.

We adopted the DNAm feature of tumor for developing a MRD assay of CRC, because of several reasons. Firstly, it was shown previously that cancer-associated methylation dominants CRC DNAm profile[^32^](#_ENREF_32). Secondly, these cancer-associated methylation signals do not only come from malignant cells but some of them directly originate from the tumor-infiltrating and activated immune cells[^33^](#_ENREF_33). In an extreme setting where eradicative therapy is applied to the patient, these immune cells might well outnumber the persistent malignant clone, serving as a proxy surrogate biomarker for detecting residual tumor presence. Thirdly, the mitotic age of cells could be directly read-out by DNA methylation.

We consider the epigenomic features of a malignant neoplasm, in its ecological environment, could be fully represented by these following features: 1. The malignant-cell-associated immune cell DNAm (M); 2. A general immune cell activation DNAm feature (I) that could further help to distinguish benign disease to malignant ones; 3. An DNAm age feature that could capture the clonal growth signal of any cell in the body; 4. The tissue-of-origin signal associated with specific type of tumor (T); and 5. The DNA fragment size information which is orthogonal to DNA methylation and associated with the abnormal epigenomic activation and cell cycle acceleration in tumors. We hence constructed, guided by such theory, a bioinformatic model *MAFIT* that integrates all these 5 features into the model to predict presence of tumor in the sample.

**Supplementary Materials and Methods**

**Study design**

The primary objective of the study was: (1) To determine the correlation between tumor fractions derived from cfDNA methylation and cfDNA somatic mutation in the surgery-naive cohort using pre-surgery blood cfDNA; (2) To determine the predictive power of ctDNA positivity by cfDNA methylation or somatic mutation for progression-free survival (PFS) as assessed by standard clinical practice including computed tomography, in patients of the post-surgery cohort using post-surgery blood cfDNA.

Donors with colorectal cancer (CRC) and planned to undergo curative-intent surgery were enrolled to the study. The analysis includes an interim population of the full study cohort, including samples (n=104) from a total of 104 donors (Table S2) separated into a surgery-naive/pre-surgery cohort and a post-surgery cohort. Routine follow-up is performed during postoperative period according to state-of-the-art clinical practical guidance including computed tomography and blood biomarker test. Records of clinical information, pathological assessment, follow-up records, and neoadjuvant/adjuvant treatments for CRC patients were collected. Routine laboratory tests and pathology assessments were done according to the relevant Chinese clinical guidelines and protocols. Post-surgery routine assessments were performed in walk-in clinic and include computed tomography at each 3-6 months. The pre-surgery cohort contains 64 pre-surgery (-30 days ~ -1 days before surgery) donors (Table S3). The post-surgery cohort contains 40 post-surgery (1 days ~ 871 days) donors (Table S6). Each donor donated one single blood draw to the study. Third-party statisticians (Dr. Minsheng Peng from a State Key Laboratory and Ms. Xinyue Cao from a Clinical Trial Center, listed as co-authors of the study) were invited to blind the samples by tracking a record between patient clinical record ID and experimental sample ID. The samples sent to lab carried only experimental sample ID. The sequencing results were provided to the statisticians. Clinical records were collected by clinical research associate and given to the statisticians. The statisticians then performed analysis according to the clinical information. A flowchart of the study including enrollment, aims and including/excluding criteria for the Challenge-CRC study cohort are indicated in Figure 1.

This study was conducted in accordance with the measures of China on the administration of clinical research and the Declaration of Helsinki. Clinical information and samples (including blood and surgical tissue specimens) of the multi-center, prospective, observational cohort for colorectal cancer: Cancer HALLmark Epigenetics aNd Genetics (*Challenge*) of CRC (named: *Challenge-CRC* study cohort) was continually and prospectively collected from 3 hospitals in 3 cities across China from 2019/01/08: Chengdu, Wuhan, Hangzhou (ethical approval number: 2017038 and 2021125). Written informed consents were obtained from all individuals enrolled in the Challenge-CRC study cohort.

**Targeted capture of somatic mutation**

Targeted capture of somatic mutation was performed as described previously[^36^](#_ENREF_36) to capture 6156 genomic regions which covered the exon/UTR of 341 oncology-relevant genes, as well as fusion hotspot introns from 17 known fusion driver genes, with an in-house hybridization assay. Briefly, >=10 ng plasma cfDNA or >=1 μg sonicated peripheral blood mononucleus cell (PBMC) genomic DNA (gDNA) are 3'-extended with terminal deoxynucleotide transferase (TdT) with poly-dT and ligated to a 3' poly-dA extruding double-stranded adaptor with T4 DNA ligase, followed by 12 rounds of linear amplification with ignition primers complement to the adaptor sequence. The linearly amplified products were ligated to a 3' poly-dN extruding double-stranded adaptor with T4 DNA ligase, and second strand synthesis was performed with Pfu, results in a double-stranded library template which could be PCR amplified. Hybridization of the amplified pre-capture library was performed with a panel of biotinylated, double-stranded DNA probes targeting 341 oncology-relevant genes.

**Targeted capture of tumor-associated DNA methylation**

The EUCAS-CRC assay (Euler Technology, China) is based on targeted capture sequencing of bisulfite-converted DNA from genomic loci differentially methylated between tissues on 18966 genomic regions. Targeted capture of tumor-associated DNA methylation was performed using a single-stranded library preparation method similar as described above. Briefly, >=6 ng plasma cfDNA were bisulfite-converted with a commercial kit (Zymo Gold), and the resulting converted single-stranded DNA are 3'-extended with poly-dT, ligated to a 3' poly-dA extruding double-stranded adaptor, linearly amplified for 12 rounds, ligated to a 3' poly-dN extruding double-stranded adaptor, PCR amplified, hybridized to a panel of biotinylated, double-stranded DNA probes targeting 2.7 Mbp of genomic regions encompassing cancer-related CpG islands.

**Estimation of tumor fraction with cfDNA somatic mutation**

Raw sequencing data of targeted captured cfDNA for somatic mutation was preprocessed by fastp, and aligned to human genome GRCh37 via BWA-mem (Sentieon), deduplicated and sorted with SAMBAMBA/SAMBLASTER. Paired cfDNA-PBMC sequencing data were passed to TNscope (Sentieon) and Pisces (Illumina) to call somatic short nucleotide variant (SNV) mutation. Concordant SNV called by both pipelines were then annotated by AnnoVAR. Only known cancer-associated pathogenic somatic mutation annotated by ClinVAR, COSMIC or with a somatic-germline Z-test score >3 in TCGA MC3 variant pool were used to calculate tumor fraction. Structural variants (SV) were called with Lumpy and only known pathogenic driver SV were included. Copy number variant (CNV) were called with CNVKit and only CNV with abs(log(CN)) >= 1.5 were included in the calculation. The copy number-derived tumor fraction was calculated via linear regression fit with a set of integer copy number for each CNV genomic region. Tumor fraction was calculated as mean variant allelic frequency (VAF) of all cancer-associated somatic mutation in cfDNA. CNV was only considered when there is no SV or SNV present in the cfDNA. If there is zero somatic mutation called by above protocol, the somatic mutation derived tumor fraction was set to zero. ctDNA positivity was determined as positive determination of a cancer-derived pathogenic somatic mutation.

**Estimation of tumor fraction with cfDNA methylation**

Raw sequencing data of targeted captured cfDNA for DNA methylation was preprocessed by fastp, and aligned to human genome GRCh37 via BWA-meth with the Sentieon BWA-mem (Sentieon), deduplicated and sorted with SAMBAMBA/SAMBLASTER. PySAM was used to process the aligned data to remove any reads containing >=3 unconverted non-CpG C base and to extract per-read CpG methylation status. For each captured genomic region, reads were categorized by the average CpG methylation level (‘methylation haplotypes’) they carried (into 10 bins from 0 to 1). Haplotype frequency was determined as mean frequency of reads of a specific methylation haplotype in all reads sequenced on a given genomic region. Tumor fraction estimated via cfDNA methylation was computed as weighted mean haplotype frequency, of all methylation haplotype which is present in CRC tumor tissue but not in PBMC or cfDNA.

**MAFIT score to estimate tumor fraction by DNA methylation**

We developed a score, MAFIT, to determine tumor fraction in cfDNA by using a combination of biomarkers. We reasoned that tumor fraction in blood cfDNA could be reflected in five independent and correlated factors: tumor-specific DNA methylation, tumor-associated immune activation, cell division age increase in the tumor clone, tumor-specific cfDNA fragmentation, and increase of cell-of-origin DNA methylation of that tumor. Hence, we utilized tumor-specific marker, immune-activation marker, cell-division-age marker, cfDNA fragment size marker, and tissue-of-origin marker in the model. MAFIT stands for M: Methylation-derived tumor fraction for CRC, A: methylation age, F: fragment size Z score, I: immune-related methylation signal, and T: tissue-of-origin specific methylation markers of each sample.

Methylation haplotypes on immune-cell-associated or cell-division-age-associated genomic regions[^35^](#_ENREF_35) were extracted as mentioned above. Tumor-associated immune cell fraction, the methylation clock age, or tissue-of-origin specific methylation signal, were computed using general linear models built with samples of known age, immune cell fraction, or given cell type on these haplotypes. Mean fragment size of sequenced cfDNA were computed and a Z-test was performed against the fragment size distribution from cfDNA sequencing data of 20 internal control individuals. Tissue-of-origin decomposition is performed by decomposing the genome-wide CpG mean beta values against mean beta values of colorectal cancer, lung cancer, upper GI cancer, breast cancer, liver cancer, and PBMC, using NNLS method.

MAFIT score is built with a general linear model with the outputs from M (estimated tumor fraction), I (estimated immune cell fraction), A (estimated methylation clock age), F (Z-score of mean fragment size), and T (a vector of tissue-of-origin output fraction from each tissue-of-origin). The predictor is built by: y ~ M + A + F + I + T. In training, y is defined as 1 (tumor patient) or 0 (non-tumor patient). Training is performed with cfDNA sequencing result from 24 CRC patients and 20 healthy donors. ctDNA positivity was determined as MAFIT > 0.5.

**Statistical analysis**

Relapse-free survival (RFS) was measured from the day of surgery to first radiographic recurrence, or definitive clinical identification of recurrence. Patients may undergo adjuvant therapy without considering ctDNA positivity as a factor. Patients were censored at the date of last follow-up, or non-cancer-related death. Linear regression and statistical significance were computed in R (3.6.2) with lm command. Test between groups were performed via Fisher's exact test (categories) or student's t-test (two group comparison), where applicable. Survival analysis was performed with survMisc and survival packages in R, using Kaplan-Meier method. A log-rank test was used for hazard ratio (HR) and all P values are based on two-sided testing, with “significance” meaning P <= 0.05. Multiple clinical cofactor analysis was performed using coxph in R.

**References**

1. Tan EM, Schur PH, Carr RI, Kunkel HG. Deoxybonucleic acid (DNA) and antibodies to DNA in the serum of patients with systemic lupus erythematosus. *J Clin Invest.* 1966;45(11):1732-1740.

2. Stroun M, Anker P, Maurice P, Lyautey J, Lederrey C, Beljanski M. Neoplastic characteristics of the DNA found in the plasma of cancer patients. *Oncology.* 1989;46(5):318-322.

3. Vasioukhin V, Anker P, Maurice P, Lyautey J, Lederrey C, Stroun M. Point mutations of the N-ras gene in the blood plasma DNA of patients with myelodysplastic syndrome or acute myelogenous leukaemia. *Br J Haematol.* 1994;86(4):774-779.

4. Sorenson GD, Pribish DM, Valone FH, Memoli VA, Bzik DJ, Yao SL. Soluble normal and mutated DNA sequences from single-copy genes in human blood. *Cancer Epidemiol Biomarkers Prev.* 1994;3(1):67-71.

5. Diehl F, Li M, Dressman D, et al. Detection and quantification of mutations in the plasma of patients with colorectal tumors. *Proc Natl Acad Sci U S A.* 2005;102(45):16368-16373.

6. Bai H, Mao L, Wang HS, et al. Epidermal growth factor receptor mutations in plasma DNA samples predict tumor response in Chinese patients with stages IIIB to IV non-small-cell lung cancer. *J Clin Oncol.* 2009;27(16):2653-2659.

7. Abbosh C, Birkbak NJ, Wilson GA, et al. Phylogenetic ctDNA analysis depicts early-stage lung cancer evolution. *Nature.* 2017;545(7655):446-451.

8. Guibert N, Hu Y, Feeney N, et al. Amplicon-based next-generation sequencing of plasma cell-free DNA for detection of driver and resistance mutations in advanced non-small cell lung cancer. *Ann Oncol.* 2018;29(4):1049-1055.

9. Malapelle U, Mayo de-Las-Casas C, Rocco D, et al. Development of a gene panel for next-generation sequencing of clinically relevant mutations in cell-free DNA from cancer patients. *Br J Cancer.* 2017;116(6):802-810.

10. Newman AM, Bratman SV, To J, et al. An ultrasensitive method for quantitating circulating tumor DNA with broad patient coverage. *Nat Med.* 2014;20(5):548-554.

11. Paweletz CP, Sacher AG, Raymond CK, et al. Bias-Corrected Targeted Next-Generation Sequencing for Rapid, Multiplexed Detection of Actionable Alterations in Cell-Free DNA from Advanced Lung Cancer Patients. *Clin Cancer Res.* 2016;22(4):915-922.

12. Phallen J, Sausen M, Adleff V, et al. Direct detection of early-stage cancers using circulating tumor DNA. *Sci Transl Med.* 2017;9(403):eaan2415.

13. Thompson JC, Yee SS, Troxel AB, et al. Detection of Therapeutically Targetable Driver and Resistance Mutations in Lung Cancer Patients by Next-Generation Sequencing of Cell-Free Circulating Tumor DNA. *Clin Cancer Res.* 2016;22(23):5772-5782.

14. Tie J, Wang Y, Tomasetti C, et al. Circulating tumor DNA analysis detects minimal residual disease and predicts recurrence in patients with stage II colon cancer. *Sci Transl Med.* 2016;8(346):346ra392.

15. Zhang X, Zhao W, Wei W, et al. Parallel Analyses of Somatic Mutations in Plasma Circulating Tumor DNA (ctDNA) and Matched Tumor Tissues in Early-Stage Breast Cancer. *Clin Cancer Res.* 2019;25(21):6546-6553.

16. Diehn M, Alizadeh AA, Adams H-P, et al. Early prediction of clinical outcomes in resected stage II and III colorectal cancer (CRC) through deep sequencing of circulating tumor DNA (ctDNA). *J Clin Oncol.* 2017;35(15_suppl):3591-3591.

17. Tie J, Cohen J, Wang Y, et al. Serial circulating tumor DNA (ctDNA) analysis as a prognostic marker and a real-time indicator of adjuvant chemotherapy (CT) efficacy in stage III colon cancer (CC). *J Clin Oncol.* 2018;36(15_suppl):3516-3516.

18. Bartak BK, Fodor T, Kalmar A, et al. A Liquid Biopsy-Based Approach for Monitoring Treatment Response in Post-Operative Colorectal Cancer Patients. *Int J Mol Sci.* 2022;23(7):3774.

19. Fischer LE, Stintzing S, Heinemann V, et al. Liquid Biopsy in Colorectal Cancer: Quo Vadis? Implementation of Liquid Biopsies in Routine Clinical Patient Care in Two German Comprehensive Cancer Centers. *Front Oncol.* 2022;12:870411.

20. Li Y, Mo S, Zhang L, et al. Postoperative circulating tumor DNA combined with consensus molecular subtypes can better predict outcomes in stage III colon cancers: A prospective cohort study. *Eur J Cancer.* 2022;169:198-209.

21. Ogaard N, Reinert T, Henriksen TV, et al. Tumour-agnostic circulating tumour DNA analysis for improved recurrence surveillance after resection of colorectal liver metastases: A prospective cohort study. *Eur J Cancer.* 2022;163:163-176.

22. Pedersen SK, Musher BL, LaPointe LC, et al. Detection of recurrent colorectal cancer with high specificity using a reporting threshold for circulating tumor DNA methylated in BCAT1 and IKZF1. *Cancer.* 2022;128(10):1921-1928.

23. Reinert T, Henriksen TV, Christensen E, et al. Analysis of Plasma Cell-Free DNA by Ultradeep Sequencing in Patients With Stages I to III Colorectal Cancer. *JAMA Oncol.* 2019;5(8):1124-1131.

24. Scholer LV, Reinert T, Orntoft MW, et al. Clinical Implications of Monitoring Circulating Tumor DNA in Patients with Colorectal Cancer. *Clin Cancer Res.* 2017;23(18):5437-5445.

25. Tie J, Cohen JD, Lahouel K, et al. Circulating Tumor DNA Analysis Guiding Adjuvant Therapy in Stage II Colon Cancer. *N Engl J Med.* 2022;386(24):2261-2272.

26. Mouliere F, Chandrananda D, Piskorz AM, et al. Enhanced detection of circulating tumor DNA by fragment size analysis. *Sci Transl Med.* 2018;10(466):eaat4921..

27. Wan JCM, Heider K, Gale D, et al. ctDNA monitoring using patient-specific sequencing and integration of variant reads. *Sci Transl Med.* 2020;12(548) :eaaz8084.

28. Zviran A, Schulman RC, Shah M, et al. Genome-wide cell-free DNA mutational integration enables ultra-sensitive cancer monitoring. *Nat Med.* 2020;26(7):1114-1124.

29. Cristiano S, Leal A, Phallen J, et al. Genome-wide cell-free DNA fragmentation in patients with cancer. *Nature.* 2019;570(7761):385-389.

30. Mathios D, Johansen JS, Cristiano S, et al. Detection and characterization of lung cancer using cell-free DNA fragmentomes. *Nat Commun.* 2021;12(1):5060.

31. Liu MC, Oxnard GR, Klein EA, Swanton C, Seiden MV, Consortium C. Sensitive and specific multi-cancer detection and localization using methylation signatures in cell-free DNA. *Ann Oncol.* 2020;31(6):745-759.

32. Hoadley KA, Yau C, Hinoue T, et al. Cell-of-Origin Patterns Dominate the Molecular Classification of 10,000 Tumors from 33 Types of Cancer. *Cell.* 2018;173(2):291-304 e296.

33. Yang R, Cheng S, Luo N, et al. Distinct epigenetic features of tumor-reactive CD8+ T cells in colorectal cancer patients revealed by genome-wide DNA methylation analysis. *Genome Biol.* 2019;21(1):2.

34. Yang Z, Wong A, Kuh D, et al. Correlation of an epigenetic mitotic clock with cancer risk. *Genome Biol.* 2016;17(1):205.

35. Horvath S. DNA methylation age of human tissues and cell types. *Genome Biol.* 2013;14(10):R115.

36. Wang Y, Ju L, Guo Z, et al. Pedigree analysis of a POLD1 germline mutation in urothelial carcinoma shows a close association between different mutation burdens and overall survival. *Cell Mol Immunol.* 2021;18(3):767-769.
